# Evaluation of blood pressure response during dobutamine stress echocardiography in patients without cardiovascular diseases

**DOI:** 10.14814/phy2.15758

**Published:** 2023-06-22

**Authors:** Leila Bigdelu, Reza Mahmoudi Meimand, Nadia Azadi, Lida Jarahi, Yoones Ghaderi, Vafa Baradaran Rahimi

**Affiliations:** ^1^ Vascular and Endovascular Surgery Research Center Mashhad University of Medical Sciences Mashhad Iran; ^2^ Department of Cardiovascular Diseases, Faculty of Medicine Mashhad University of Medical Sciences Mashhad Iran; ^3^ Department of Community Medicine, Faculty of Medicine Mashhad University of Medical Sciences Mashhad Iran; ^4^ Pharmacological Research Center of Medicinal Plants Mashhad University of Medical Sciences Mashhad Iran

**Keywords:** diastolic blood pressure, dobutamine stress echocardiography, systolic blood pressure

## Abstract

Dobutamine stress echocardiography (DSE) is a diagnostic tool for determining coronary artery disease. Considering hypotension and hypertension as important complications of DSE, we aimed to evaluate the blood pressure (BP) responses during DSE. Patients without known cardiovascular diseases who underwent DSE were included. We excluded patients who had hypertension, diabetes mellitus, a known history of cardiovascular diseases, and those taking vasoactive medications. Systolic (SBP) and diastolic (DBP) blood pressure were recorded at baseline and peak stress. We included 688 patients with an age of 57.9 ± 12.01 years. During DSE, SBP (+19.72 ± 26.51 mm Hg, *p <* 0.001), DBP (+5.52 ± 17.35 mm Hg, *p <* 0.001), and HR (+54.05 ± 22.45 bpm, *p <* 0.001) significantly increased from baseline to peak stress. The normal cut‐off value was measured between 101–210 mm Hg for SBP and 50–121 mm Hg for DBP. According to this normal cutoff, 11 (1.3%) and 30 (4.4%) patients had hypotensive and hypertensive SBP and 15 (2.2%) and 21 (3.1%) patients had hypotensive and hypertensive DBP, respectively. The hypotensive response was correlated with baseline SBP (*r* = 0.6, *p* = 0.001) and atropine (*r* = −2.18, *p* = 0.043), and the hypertensive response was correlated with baseline SBP (*r* = 0.048, *p <* 0.001). Baseline BP and atropine consumption were the independent variables associated with the outside‐the‐normal range of blood pressure responses.

## 1 INTRODUCTION

Dobutamine stress echocardiography test (DSE) is currently utilized as a diagnostic and prognostic tool for patients suspected to have or those who have established coronary artery disease (CAD) (Marwick, 2018; Pellikka et al., 2007). In most patients, DSE is well‐tolerated, and it is generally accepted as a safe method. However, DSE may be associated with a number of serious complications. Abnormal blood pressure (BP) responses are one the most common complications that may result in early discontinuation of the test. Although DSE has been used for over two decades, the patterns of BP changes are not well‐studied. A study with a large sample size showed increased systolic BP and decreased diastolic BP (Abram et al., 2016). Another similar study showed normal, hypertensive, and hypotensive BP responses in 90%, 9%, and 1% of the cases, respectively (Abram et al., 2017). In previous studies, different cutoff values have been used for describing abnormal BP responses during DSE (Mathias Jr. et al., 1999; Lee et al., 1997; Sorrentino et al., 1996; Cortigiani et al., 2002). Furthermore, these studies have included patients with underlying diseases such as diabetes, hypertension, or cardiovascular diseases. These conditions and treatments can potentially change BP responses during DSE and its findings. Unfortunately, there is still no consensus on the normal values of BP during DSE.

This study aimed to evaluate the normal pattern of BP responses during DSE and define cutoff points for normal systolic (SBP) and diastolic (DBP) responses.

## 2 MATERIALS AND METHODS

### 2.1 Ethics

This study was confirmed by the ethics committee of Mashhad University of Medical Sciences (approval code. IR.MUMS.fm.REC.1395.465). All participants received and signed written informed consent.

### 2.2 Study protocol

We included patients who underwent DSE at Qaem Hospital, affiliated with Mashhad University of Medical Sciences, Mashhad, Iran. The patient's demographic and clinical characteristics were extracted from their medical documents. In addition, the patient's baseline and peak stress BP and heart rate were recorded. Patients with hypertension, diabetes mellitus, taking anti‐hypertensive drugs, and a history of cardiovascular diseases such as prior myocardial infarction, coronary artery bypass graft, valvular diseases, and percutaneous coronary revascularization were excluded.

### 2.3 DSE protocol

Dobutamine was intravenously administered by an infusion pump with an initiating dose of 10 μg.kg.min. After 3 min, the dobutamine dose was increased to 20 μg/kg/min. Then its dose was increased every 3 min to reach the maximum dose of 40 μg/kg/min if necessary. Atropine was administered intravenously in patients who did not achieve their target heart rate, which was 85% of the age‐related maximal heart rate. Atropine was started with a dose of 0.25 mg and was incremented every 1 min to a maximum dose of 2 mg if needed. In accordance with the guideline, we terminated the test in case of severe hypertension (SBP > 240 mm Hg and DBP > 120 mm Hg) and hypotension (SB*P <* 90 mm Hg in association with symptoms) (Fazlinezhad et al., 2014; Poorzand et al., 2021).

### 2.4 BP evaluation

A specially trained nurse measured SBP and DBP in patients with the left lateral decubitus position. BP was measured at the baseline, every 3 min during the stress test, at peak, and in recovery (Gholoobi et al., 2021). A physician confirmed the BP results. ΔSBP was defined as the difference between SBP at peak stress and SBP at baseline. Furthermore, ΔDBP was calculated as the difference between baseline DBP and DBP at peak stress.

### 2.5 Echocardiography

A two‐dimensional echocardiography was performed at baseline, during the stress test, and in recovery. The left ventricular ejection fraction was measured by visual assessment or the Simpsons method. The wall motion was examined by the 16 segments model, which the American Society of Echocardiography has suggested (Bigdelu et al., 2022; Mohammadi et al., 2022).

### 2.6 Estimation of sample size

The level of significance (α) was set to 5%, $Z_{1-\frac{\alpha }{2}}$ = 1.96, d= 0.018, and p= 0.06. Based on a previous study (Abram et al., 2016), according to the formula for the proportion of the qualitative trait, the required sample size was 670.

$$N=\frac{Z_{1-\frac{\alpha }{2}}\times P\left(1-P\right)}{d^{2}}$$

### 2.7 Statistical analysis

The SPSS statistical program version.16 (SPSS Inc., Chicago, Illinois) was used to analyze the data. Parametric data were expressed as Means and standard deviations (SD), whereas non‐parametric outcomes were expressed as a number (*n*) with a percentage (%). The comparison between BP levels during DSE was performed using paired sample t‐test or Wilcoxon test. In addition, the *t*‐test and Tukey or Mann–Whitney *U* and Kruskal–Wallis test evaluated the difference between BP changes according to age, gender, and atropine usage. Furthermore, a logistic regression analysis was performed to evaluate predictors of hypertensive and hypotensive responses during DSE.

Cutoff values were defined according to peak SBP and DBP. In addition, the upper and lower BP limits were defined regarding adding subtracting 2SD. The significance level of 0.05 was considered statistically significant.

## 3 RESULTS

Totally, we evaluated 688 patients without known cardiovascular diseases who underwent DSE in our study. The mean age of the patients was 57.79 ± 12.01 years old; 60.9% (419) were female, and 39.1% were male (Table 1).

**TABLE 1:** Demographic and anthropometric data of patients enrolled in the study.

Variable (*N* = 688)	Min–max	Mean ± SD or *N* (%)
Age (Years)	28–99	57.79 ± 12.01
Height (cm)	133–186	157.65 ± 9.88
Weight (Kg)	34–118	69.05 ± 14.55
Gender	Male	269 (39.1%)
	Female	419 (60.9%)
Age category	<50	169 (24.6%)
	50–59	237 (34.4%)
	60–69	158 (23.0%)
	≥70	124 (18.0%)

### 3.1 Changes of SBP, DBP, and HR during DSE

**FIGURE 1:**
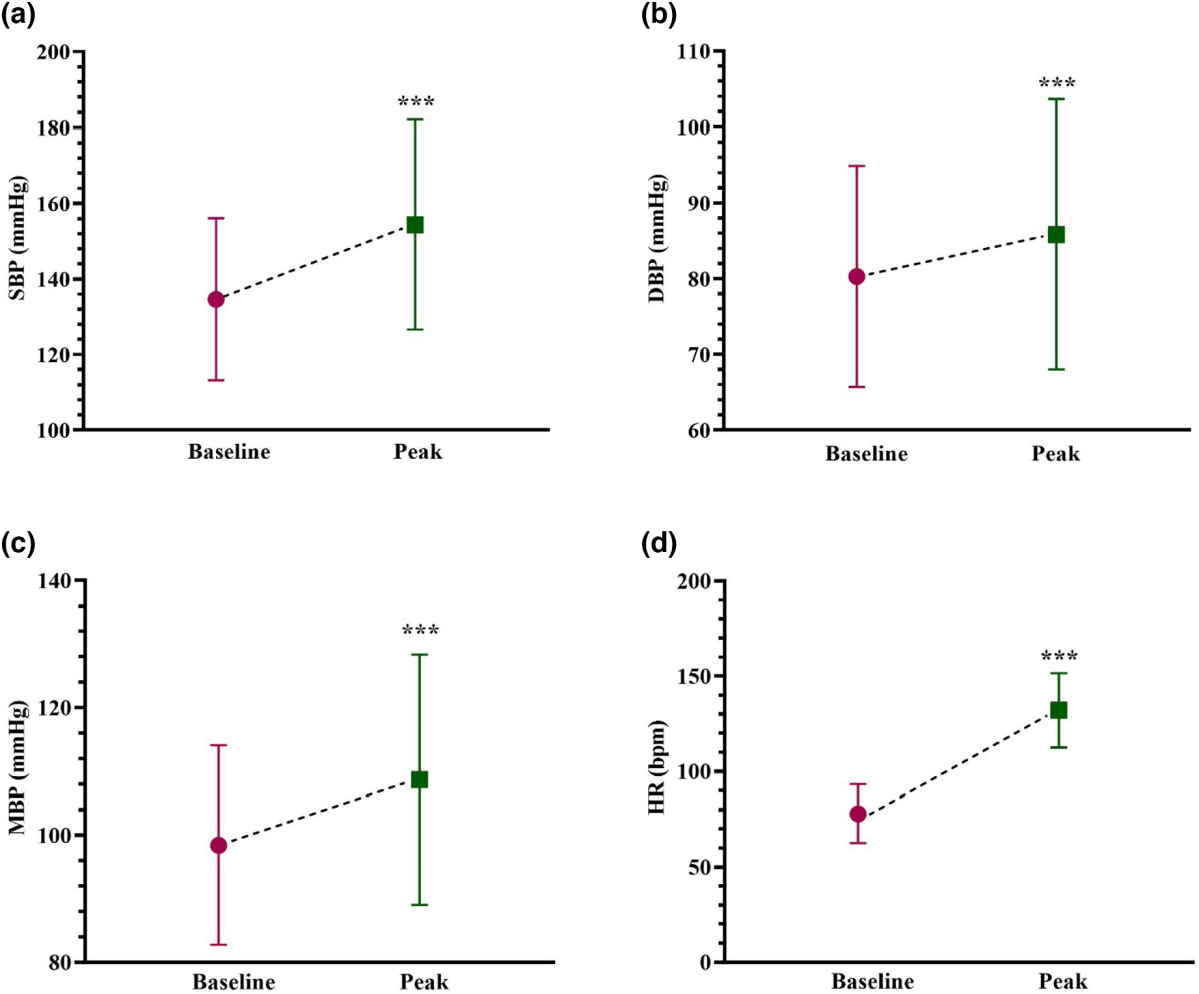
Blood pressure and heart rate responses during DSE; BDP, diastolic blood pressure; HR, heart rate; MBP, mean blood pressure; SBP, Systolic blood pressure; comparing the results of baseline and peak stress using Paired *t*‐test; ***p < 0.001.

During DSE, SBP (19.72 ± 26.51 mm Hg, *p <* 0.001), DBP (5.52 ± 17.35 mm Hg, *p <* 0.001), mean BP (10.25 ± 18.61 mm Hg, *p <* 0.001), and heart rate (HR) (54.05 ± 22.45 bpm, *p <* 0.001) meaningfully propagated from baseline to the peak stress (Figure 1).

**FIGURE 2:**
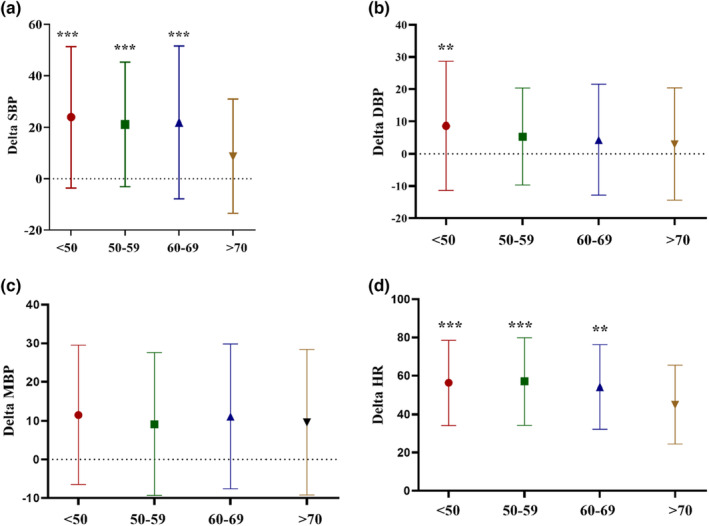
Delta blood pressure and heart rate responses during DSE; BDP, diastolic blood pressure; HR, heart rate; MBP, mean blood pressure; SBP, Systolic blood pressure; comparing the results of different age groups using one‐way ANOVA‐test and Tukey post‐hoc test, ***p < 0.001 and **p < 0.01 comparing the respected group and > 70 group.

We also stratified BP responses according to age, as shown in Table 2. Our results revealed that the most changes in SBP (23.86 ± 27.46 mm Hg) and DBP (8.67 ± 20.05 mm Hg) during DSE were observed in patients under 50 years old. In contrast, the lowest changes in SBP (8.69 ± 22.15 mm Hg) and DBP (3.08 ± 17.38 mm Hg) were recorded in patients over 70 years old. In addition, delta SBP, delta DBP, and delta HR were remarkably mitigated with increasing age (*p <* 0.001, *p* = 0.032, and *p <* 0.001, respectively, Table 2, Figure 2). Moreover, delta SBP, delta DBP, and delta HR was notably higher in patients under 50 years old compared to over 70 years old (*p <* 0.001 and *p <* 0.01, Figure 2).

**TABLE 2:** Evaluation of blood pressure and heart rate responses during DSE according to age category.

Age (years)	<50 *N* = 169	50–59 *N* = 237	60–69 *N* = 124	≥70 *N* = 158	*p*‐value^a^
Baseline SBP (mm Hg)	132.78 ± 22.28	132.55 ± 21.27	136.97 ± 20.25	138.07 ± 21.69	**0.035** *
Peak SBP (mm Hg)	156.64 ± 30.59	153.63 ± 23.2	158.89 ± 30.82	146.77 ± 25.92	**0.002** **
Delta SBP (mm Hg)	23.86 ± 27.46	21.08 ± 24.19	21.91 ± 29.72	8.69 ± 22.15	**<0.001** ***
Baseline DBP (mm Hg)	82.18 ± 16.37	80.61 ± 14.21	80.47 ± 12.78	76.95 ± 14.59	**0.024** *
Peak DBP (mm Hg)	90.85 ± 21.76	85.94 ± 13.6	84.82 ± 17.54	80.04 ± 17.56	**<0.001** ***
Delta DBP (mm Hg)	8.67 ± 20.05	5.32 ± 15	4.35 ± 17.18	3.08 ± 17.38	**0.032** *
Baseline MBP (mm Hg)	96.02 ± 14.32	100.9 ± 16.62	96.29 ± 15.15	99.6 ± 15.35	**0.003** **
Peak MBP (mm Hg)	107.55 ± 20.06	110.03 ± 19.73	107.43 ± 20.13	109.18 ± 18.36	**0.496**
Delta MBP (mm Hg)	11.52 ± 18.06	9.12 ± 18.45	11.13 ± 18.74	9.57 ± 18.85	**0.538**
Baseline HR (mm Hg)	81.94 ± 16.09	77.43 ± 13.42	75.24 ± 16.62	76.75 ± 15.15	**0.001** ***
Peak HR (mm Hg)	138.26 ± 21.27	134.51 ± 20.39	129.5 ± 15.21	121.72 ± 15.05	**<0.001** ***
Delta HR (mm Hg)	56.31 ± 22.17	57.07 ± 22.8	54.25 ± 22	44.96 ± 20.53	**<0.001** ***

^a^ Comparing the results of different age ranges groups using One‐way ANOVA.

* *p* < 0.05.

** *p* < 0.01.

*** *p* < 0.001.

Abbreviations: BDP, diastolic blood pressure; HR, Heart rate; SBP, Systolic blood pressure.

There were no significant differences between SBP, DBP, and HR responses between male and female patients (*p* > 0.05, Table 3).

**TABLE 3:** Evaluation of blood pressure and heart rate responses during DSE according to gender.

Variable	Male *N* = 269	Female *N* = 419	*p*‐value^a^
Baseline SBP (mm Hg)	132.89 ± 19.77	135.73 ± 22.43	**0.091**
Peak SBP (mm Hg)	154.96 ± 28.44	153.94 ± 27.26	**0.638**
Delta SBP (mm Hg)	22.07 ± 28.17	18.21 ± 25.32	**0.063**
Baseline DBP (mm Hg)	80.13 ± 15.09	80.41 ± 14.3	**0.806**
Peak DBP (mm Hg)	86.62 ± 18.01	85.32 ± 17.69	**0.352**
Delta DBP (mm Hg)	6.48 ± 17.72	4.9 ± 17.1	**0.245**
Baseline MBP (mm Hg)	98.62 ± 15.58	98.27 ± 15.69	**0.777**
Peak MBP (mm Hg)	109.35 ± 20.32	108.23 ± 19.23	**0.465**
Delta MBP (mm Hg)	10.72 ± 17.93	9.95 ± 19.06	**0.594**
Baseline HR (mm Hg)	76.6 ± 15.86	78.75 ± 14.96	**0.073**
Peak HR (mm Hg)	131.74 ± 20.7	132.12 ± 18.67	**0.801**
Delta HR (mm Hg)	55.13 ± 23.29	53.36 ± 21.9	**0.315**

The significance level of 0.05 was considered statistically significant (bold).

^a^ Comparing the results of baseline and peak stress using Independent samples *t*‐test.

Abbreviations: BDP, diastolic blood pressure; HR, Heart rate; SBP, Systolic blood pressure.

Overall, 228 patients received atropine. In order to achieve target heart rate, atropine was administrated to 66% of patients under 50 years old, 53% of patients between 50 and 59 years, 37% of patients between 60 and 69 years, and 23% of patients over 70 years old. Patients who received atropine had meaningfully higher delta SBP, delta DBP, and delta HR than those who did not receive atropine (*p <* 0.001 for all cases, Table 4).

**TABLE 4:** Evaluation of blood pressure and heart rate responses during DSE according to atropine usage.

Variable	Atropine received *N* = 228	Not‐atropine received *N* = 460	*p*‐value^a^
Baseline SBP (mm Hg)	129.43 ± 22.39	137.19 ± 20.52	**<0.001** ***
Peak SBP (mm Hg)	157.11 ± 27.47	152.97 ± 27.75	**0.065**
Delta SBP (mm Hg)	27.68 ± 27.09	15.77 ± 25.34	**<0.001** ***
Baseline DBP (mm Hg)	77.36 ± 14.94	81.76 ± 14.22	**<0.001** ***
Peak DBP (mm Hg)	89.1 ± 17.3	84.21 ± 17.86	**0.001** ***
Delta DBP (mm Hg)	11.73 ± 17.47	2.44 ± 16.46	**<0.001** ***
Baseline MBP (mm Hg)	97.01 ± 16.13	99.1 ± 15.36	**0.099**
Peak MBP (mm Hg)	107.91 ± 20.42	109.04 ± 19.28	**0.476**
Delta MBP (mm Hg)	10.89 ± 20	9.93 ± 17.9	**0.526**
Baseline HR (mm Hg)	71.88 ± 12.34	80.9 ± 15.81	**<0.001** ***
Peak HR (mm Hg)	133.1 ± 21.53	131.42 ± 18.37	**0.288**
Delta HR (mm Hg)	61.21 ± 23.04	50.51 ± 21.31	**<0.001** ***

^a^ Comparing the results of baseline and peak stress using Independent samples *t*‐test.

*** *p* < 0.001.

Abbreviations: BDP, diastolic blood pressure; HR, Heart rate; SBP, Systolic blood pressure.

### 3.2 Evaluation of the cutoff points for normal SBP and DBP changes during DSE

In order to define normal BP responses during DSE, we used mean BP ± 2SD. Normal SBP and DBP cut‐point values were defined between 101–210 mm Hg and 50–121 mm Hg, respectively (Table 5). In addition, the normal cutoff points for SBP and DBP in different age ranges are illustrated in Table 5.

**TABLE 5:** Evaluation of normal cutoff points for SBP and DBP changes during DSE.

Age (years)	<50	50–59	60–69	≥70	Total
Peak SBP (mm Hg)	156.64 ± 30.59	153.63 ± 23.2	158.89 ± 30.82	146.77 ± 25.92	154.34 ± 27.71
+2SD	217.82	200.03	220.53	198.61	209.92
−2SD	95.46	107.23	97.25	94.93	100.92
Peak DBP (mm Hg)	90.85 ± 21.76	85.94 ± 13.6	84.82 ± 17.54	80.04 ± 17.56	85.83 ± 17.82
+2SD	134.37	113.14	119.9	115.16	121.47
−2SD	47.33	58.74	49.74	42.92	50.19

Abbreviations: BDP, diastolic blood pressure; SBP, Systolic blood pressure; SD, Standard deviation.

Regarding our findings, we found 647 (94%) had normal SBP responses, while 11 patients (1.6%) had hypotensive, and 30 patients (4.4%) had hypertensive SBP responses. Moreover, 652 patients (94.8%) had normal DBP responses, while hypotensive and hypertensive DBP responses occurred in 15 patients (2.2%) and 21 patients (3.1%), respectively.

### 3.3 Association of hypotensive and hypertensive responses during DSE with age, gender, baseline SBP, and atropine using logistic regression

Logistic regression showed that hypotensive response is significantly associated with baseline SBP (B = 0.6, SE = 0.018, and *p* = 0.001) and atropine usage (B = −2.18, SE = 1.07, and *p* = 0.043). Furthermore, baseline SBP was the only predictor for hypertensive response (B = 0.048, SE = 0.009, and *p <* 0.001, Table 6).

**TABLE 6:** Association of hypotensive and hypertensive responses during DSE with variables using logistic regression.

Dependent variable	Independent variable	B	Standard error	OR	95% CI for OR	*p*‐Value
	Age	−0.027	0.024	0.973	0.928–1.02	0.252
	Gender	1.07	0.649	2.93	0.8234–10.48	0.097
Hypotensive response	Baseline SBP	0.06	0.018	1.06	1.02–1.1	0.001
	Atropine usage	−2.18	1.07	0.11	0.937–0.937	0.043
	Age	−0.023	0.017	0.978	0.946–1.01	0.181
Hypertensive response	Gender	−0.057	0.414	0.944	0.42–2.12	0.89
	Baseline SBP	0.048	0.009	1.04	1.3–1.6	<0.001
	Atropine usage	−0.31	0.415	0.733	0.325–1.65	0.455

Abbreviations: BDP, diastolic blood pressure, CI, confidence interval; HR, heart rate; OR, odds ratio; SBP, systolic blood pressure.

## 4 DISCUSSION

The present study assessed BP responses during DSE among patients without known cardiovascular diseases and not taking vasoactive medication. Our study showed that these patients experienced a significant increase in SBP, DBP, and HR during DSE. In addition, SBP, DBP, and HR significantly increased in younger patients and those who received atropine. According to our measured normal cutoff, 11 (1.3%) and 30 (4.4%) patients had hypotensive and hypertensive SBP, and 15 (2.2%) and 21 (3.1%) patients had hypotensive and hypertensive DBP, respectively. In addition, the hypotensive response was correlated with baseline SBP and atropine, and the hypertensive response was only associated with baseline SBP.

Dobutamine possesses sympathomimetic effects and predominantly stimulates beta 1 and 2 receptors with minimal alfa‐adrenergic effects. Dobutamine administration increases myocardial oxygen demand through its agonistic effects on beta‐1 receptors of the heart (Sachdeva & Paul, 2016). Although dobutamine mainly affects the myocardium, it is also associated with weaker effects on peripheral vessels. It is also a beta‐2 adrenergic receptor agonist resulting in vasodilation. Moreover, it causes a mild increase in heart rate by baroreflex‐mediated mechanism. In our study, dobutamine induced increased myocardial contraction and acted as a vasopressor with the clinical presentation of a significant increase in SBP, DBP, and HR. The results of this study are consistent with previous studies. In line with our results, Tadamura et al. demonstrated that the co‐administration of dobutamine and atropine increased myocardial blood flow, SBP, and DBP (Tadamura et al., 2001). Clinical studies have also reported the different levels of upregulation in SBP (Biagini et al., 2005; Cortigiani et al., 2002) and DBP (Cortigiani et al., 2002) during DSE. However, the results of these studies are not comparable with our results because they included patients with cardiovascular‐related comorbidities. Besides this, these patients take vasoactive medications, affecting cardio‐vascular responses to dobutamine‐induced stress. In this regard, patients with hypertension usually have increased vascular resistance. Therefore, dobutamine‐induced vasoconstriction may not be balanced by prompt vasodilation. Moreover, hypertensive patients have hypersensitivity of the sympathetic nervous system, which worsens the effects of dobutamine on alpha‐1 adrenergic receptors.

The present study measured the normal cutoff value between 101–210 mm Hg for SBP and 50–121 mm Hg for DBP during DSE. According to this normal cutoff, 11 (1.3%) and 30 (4.4%) patients had hypotensive and hypertensive SBP and 15 (2.2%) and 21 (3.1%) patients had hypotensive and hypertensive DBP, respectively. Similarly, Abram et al. supported the normal cutoff value between 82–182 mm Hg for SBP and 40–96 mm Hg for DBP during DSE (Abram et al., 2016). In addition, Lee and coworkers investigated the BP responses of 3129 patients during DSE and reported a hypertensive SBP of more than 220 mm Hg and hypertensive DBP of more than 110 mm Hg. According to their normal cutoff value, 30 patients (1%) had hypertensive responses during DSE (Lee et al., 1997). The results of these studies are in line with our results regarding the changes in BP during DSE.

Our findings showed that BP changes during DSE were greater in men but were not statistically significant. There is no consensus on the effect of gender on BP response during DSE. Similar to our findings, Biagini et al. showed no significant difference in SBP between men and women (Biagini et al., 2005). However, Abram and coworkers presented that men had greater delta SBP and lower delta DBP than women during DSE (Abram et al., 2016). A similar study reported that gender influences BP response and HR (Tsutsui et al., 2007). However, the exact mechanism responsible for this difference is not entirely clear, but it may be related to the difference in mean arterial pressure (Mancia et al., 2014).

In this study, the changes in BP during DSE were significantly greater in younger patients and decreased with age. Our results are similar to previous studies (Chenzbraun et al., 1999; Poldermans et al., 1995). There is no unique explanation for the role of gender in BP response, and it seems that many factors are contributing. First, senile changes in baroreflexes responses lead to decreased HR elevation in response to stress (Docherty, 1990; Lakatta, 1990). Second, aging is associated with increased vascular stiffness, resulting in lower BP change levels (Laurent et al., 2006).

## 5 CONCLUSION

The normal cutoff value was measured between 101–210 mm Hg for SBP and 50–121 mm Hg for DBP during DSE. According to this normal cutoff, 11 (1.3%) and 30 (4.4%) patients had hypotensive and hypertensive SBP and 15 (2.2%) and 21 (3.1%) patients had hypotensive and hypertensive DBP, respectively. In addition, the hypotensive response was correlated with baseline SBP and atropine, and the hypertensive response was only associated with baseline SBP. Determining the range of BP changes during DSE and its related factors may be beneficial to differentiate between normal and abnormal BP responses in DSE.

## AUTHOR CONTRIBUTIONS

Leila Bigdelu: Conceptualization, Methodology, Funding Acquisition, Investigation; Reza Mahmoudi Meimand: Investigation, Data Curation; Nadia Azadi: Investigation, Writing—Original Draft; Lida Jarahi: Formal Analysis, Data Curation; Yoones Ghaderi: Investigation, Writing—Original Draft; Vafa Baradaran Rahimi: Formal Analysis, Writing—Original Draft, Writing—review & editing.

## FUNDING INFORMATION

This study was financially supported by the research council of Mashhad University of Medical Sciences (Grant Number: 950710).

## CONFLICT OF INTEREST STATEMENT

There is no conflict of interest.

## ETHICS STATEMENT

This study was confirmed by the ethics committee of Mashhad University of Medical Sciences (approval code. IR.MUMS.fm.REC.1395.465). All participants received and signed written informed consent.

## Data Availability

Data are available from the corresponding author upon reasonable request.
